# Pharmacogenomics of Antidepressant and Antipsychotic Treatment: How Far Have We Got and Where Are We Going?

**DOI:** 10.3389/fpsyt.2020.00094

**Published:** 2020-03-12

**Authors:** Roos van Westrhenen, Katherine J. Aitchison, Magnus Ingelman-Sundberg, Marin M. Jukić

**Affiliations:** ^1^Department of Psychiatry, Parnassia Group, Amsterdam, Netherlands; ^2^Department of Psychiatry and Neuropsychology, Faculty of Health, Medicine and Life Sciences, Maastricht University, Maastricht, Netherlands; ^3^Departments of Psychiatry and Medical Genetics, University of Alberta, Edmonton, AB, Canada; ^4^Pharmacogenetics Section, Department of Physiology and Pharmacology, Karolinska Institutet, Stockholm, Sweden; ^5^Department of Physiology, Faculty of Pharmacy, University of Belgrade, Belgrade, Serbia

**Keywords:** CYP2C19, CYP2D6, schizophrenia, depression, genotyping, NGS

## Abstract

In recent decades, very few new psychiatric drugs have entered the market. Thus, improvement in the use of antidepressant and antipsychotic therapy has to focus mainly on enhanced and more personalized treatment with the currently available drugs. One important aspect of such individualization is emphasizing interindividual differences in genes relevant to treatment, an area that can be termed neuropsychopharmacogenomics. Here, we review previous efforts to identify such critical genetic variants and summarize the results obtained to date. We conclude that most clinically relevant genetic variation is connected to phase I drug metabolism, in particular to genetic polymorphism of *CYP2C19* and *CYP2D6*. To further improve individualized pharmacotherapy, there is a need to take both common and rare relevant mutations into consideration; we discuss the present and future possibilities of using whole genome sequencing to identify patient-specific genetic variation relevant to treatment in psychiatry. Translation of pharmacogenomic knowledge into clinical practice can be considered for specific drugs, but this requires education of clinicians, instructive guidelines, as well as full attention to polypharmacy and other clinically relevant factors. Recent large patient studies (n > 1,000) have replicated previous findings and produced robust evidence warranting the clinical utility of relevant genetic biomarkers. To further judge the clinical and financial benefits of preemptive genotyping in psychiatry, large prospective randomized trials are needed to quantify the value of genetic-based patient stratification in neuropsychopharmacotherapy and to demonstrate the cost-effectiveness of such interventions.

## Introduction

Despite intensive effort in neuroscience research, very few new psychopharmacological agents have entered the market during recent decades. Antidepressants in general aim to increase monoaminergic neurotransmission by blocking monoamine reuptake, while antipsychotics mostly aim to reduce mesolimbic dopaminergic neurotransmission by blocking receptors including D_2_ and 5-HT_2A_ receptors (1). However, these effects are neither necessary nor sufficient for a favorable treatment response and the effectiveness of therapy is therefore suboptimal. Current selection of an appropriate antipsychotic or antidepressant drug to a great extent still relies on psychiatrists’ clinical experience as well as on a potentially long trial and error approach, with potential serious adverse reactions such as suicidal ideation and behaviors. Moreover, while antidepressants and antipsychotics are superior compared to placebo (2, 3), their efficacy is not impressive keeping in mind the effect size of superiority over placebo (4). Since molecular targets for psychiatric drugs are not yet fully elucidated, many of the currently available drugs will likely remain the cornerstone of pharmacotherapy in psychiatry for the foreseeable future. It is therefore of paramount value to maximize their effectiveness prospectively by treatment personalization.

In general, differences between individuals in drug treatment response can be caused by environmental, physiological, and psychological factors, as well as by comorbidities and genetic variability (5, 6). With respect to the genetic component, one can estimate that roughly one quarter of the total variability in drug response is genetic in origin (7). To facilitate the transfer of genetic information to physicians, regulatory agencies incorporate pharmacogenomic drug labels into the summaries of product information (SmPC); here they specify which of the genetic variation information is important to consider with regard to drug prescription. Such drug labels include mandatory guidelines, recommendations and information about how pharmacogenomic variation should be taken into account regarding the indication or dosage - data that are expected to be beneficial for individualization of drug therapy (6). In addition two different organizations, CPIC (Clinical Pharmacogenetics Implementation Consortium) and DPWG (Dutch Pharmacogenetics Working Group) publish similar guidance based on their own expertise and judgment. It was published recently however, that the concordance among pharmacogenomic drug labels between FDA, EMA, DPGW, and CPIC is low and of drugs considered by all agencies; for only 18% of them is the information similar across the four agencies (8). For pharmacogenomic drug labels provided by the FDA and the EMA (or the Dutch or the German MPAs), the concordance is low (8, 9), in contrast to the high concordance between the FDA and the EMA regarding the approval of new drugs (10). Furthermore, the use of such labels in a clinical setting is relatively limited apart from in oncology (11).

There is substantial interindividual variability in the response and efficacy of CNS active drugs and it has become evident that genes encoding pharmacokinetic-related biomolecules have significant impact (https://cpicpgx.org/guidelines/). In particular, functional polymorphisms in genes encoding the drug metabolizing enzymes CYP2C19 and CYP2D6 are quite frequent among all populations (12) and these variants are associated with altered drug exposure sufficiently to support the clinical utility of *CYP2C19* and *CYP2D6* genotyping (13–15). In contrast, receptors that are currently used as drug targets for psychiatric drugs are evolutionary conserved to a higher extent than genes encoding drug metabolism and the actionability of pharmacodynamic-related genotyping is currently still questionable (16). However, when the functional interpretation of common or rare variants in such genes becomes available, it is clear that such pharmacogenomic information can be used to improve pharmacotherapy individualization (17).

Many findings to date in the field of pharmacogenomics in psychiatry have lacked consensus and yielded a lot of controversy. We herein review the most important studies in the field and summarize the current situation, outline future directions, and discuss possible implementation of genetic biomarkers in psychiatry with a particular focus on the *CYP2C19* and *CYP2D6* genes.

### Biomarkers Based on Genes Coding Drug Metabolizing Enzymes

In phase I, drugs are usually transformed by oxidation, demethylation, reduction, or hydrolysis to more soluble compounds, which facilitates their subsequent elimination from the body. A major phase I enzyme family is the cytochrome P450s (CYPs), whose activity usually leads to the reduction of drug potency. The human liver possesses a wide spectrum of CYP isoforms; the most abundant isoforms (CYP1A2, CYP2C9, and CYP3A4/5) (18) account for more than half of total CYP content in the human liver and they participate in metabolism of roughly one third of psychiatric drugs (19). Importantly, certain drugs are known to induce or inhibit these enzymes and consequently, polypharmacy can affect the exposure of many psychiatric drugs (https://drug-interactions.medicine.iu.edu/MainTable.aspx). In addition, one recently published and adequately powered study suggests that the *CYP1A2* SNP rs2472297 may predict clozapine exposure (20) and potentially affect clozapine treatment. However, at this point, CYP2C19 and CYP2D6 enzymes seem to be more important for pharmacogenetics in psychiatry, since they contribute significantly to the phase I metabolism of more than two thirds of all currently available psychiatric drugs (19). Whilst CYP2C19 and CYP2D6 are much less abundantly expressed in the human liver than the other CYPs mentioned above, they seem to have a very high affinity for the molecular structures on which most of the currently available psychiatric drugs are based. However, unlike the major form of hepatic CYP3A P450 isoform CYP3A4 (21), the *CYP2D6* and *CYP2C19* genes are highly polymorphic, and this genetic variation is associated with profound changes in enzymatic capacity (**Tables 1** and **2**). Owing to this genetic variability with demonstrated clinical relevance, all currently commercially available pharmacogenetic-based decision-support tools in psychiatry encompass the common variants in the *CYP2C19* and *CYP2D6* genes (13). However, in order to appropriately personalize treatment based on *CYP2C19/CYP2D6* genotype in psychiatry, prescribers need to know (i) how and in what depth *CYP2C19/CYP2D6* genotyping should be performed, (ii) the relationship between genotypic variation and the relevant linked phenotypes, and (iii) how to appropriately use this information to improve pharmacotherapy. While admirable progress in this regard has been made to date, certain research and clinical application gaps still remain to be addressed.

**Table 1:** Relation between genotype and phenotype among diploid genotypes of CYP2C19 and CYP2D6.

CYP2C19			
Genotype	Functional Diplotype	Categorization	Enzymatic capacity
*CYP2C19Null/Null*	PM/PM	Poor	0%
*CYP2C19Null/Wt*	PM/NM	Intermediate	50%
CYP2C19Null/*17	PM/UM	Intermediate	60%
*CYP2C19Wt/Wt*	NM/NM	Normal	100%
CYP2C19Wt/*17	NM/UM	Ultrarapid	110%
CYP2C19*17/*17	UM/UM	Ultrarapid	120%
**CYP2D6**			
Genotype	Functional Diplotype	Categorization	Enzymatic capacity
*CYP2D6Null/Null*	PM/PM	Poor	0%
CYP2D6Null/*41	PM/IM	Intermediate	5%
CYP2D6Null/*9-10	PM/IM	Intermediate	15%
CYP2D6*41/*9-10	IM/IM	Intermediate OR Normal	20%
CYP2D6*9-10/*9-10	IM/IM	Intermediate OR Normal	30%
*CYP2D6Wt/Null*	NM/PM	Intermediate OR Normal	50%
CYP2D6Wt/*41	NM/IM	Normal	55%
CYP2D6Wt/*9-10	NM/IM	Normal	65%
*CYP2D6Wt/Wt*	NM/NM	Normal	100%
*CYP2D6WtX3*	UM/UM	Ultrarapid	150%

NM, normal metabolizer; PM, poor metabolizer; IM, intermediate metabolizer; UM, ultrarapid metabolizer. The definition of the IM phenotype for CYP2D6 is different between sources. Enzymatic capacities are based on *in vivo* data from the recent three large-scale clinical studies (14, 15, 22). Enzymatic capacities may be substrate dependent.

**Table 2:** world-wide frequencies of common variant *CYP2C19* and *CYP2D6* alleles.

Allele	Europeans	Africans	East-Asians	South-Asians	Americans
CYP2C19*2	**18.3**	**18.1**	**31.0**	**34.0**	**10.1**
CYP2C19*3	rare	rare	**6.7**	rare	rare
CYP2C19*17	**22.4**	**23.5**	1.5	**13.6**	**12.0**
*CYP2D6xN* *Amplification*	2.3	**9.3**	2	1.5	1
CYP2D6*3	4.1	rare	rare	rare	rare
CYP2D6*4	**15.5**	**11.9**	rare	**11.6**	**15.7**
CYP2D6*5 *Deletion*	3	4	**6.5**	2	3
CYP2D6*6	2.2	rare	rare	rare	rare
CYP2D6*9	1.6	rare	rare	rare	1.3
CYP2D6*10	rare	3.2	**58.7**	**6.5**	rare
CYP2D6*17	rare	**19.7**	rare	rare	1
CYP2D6*29	rare	**9.2**	rare	rare	rare
CYP2D6*41	3.0	3.0	3.0	**13.5**	3.5

Frequencies are shown in percentages; the table is adapted from Zhou et al., 2017. Only variants with minor allelic frequencies (MAF) > 1% in any of the populations are listed in this table. The MAF < 1% variants are annotated as “rare,” the 1% < MAF < 5% variants were represented with the MAF in percentages, and the variants with MAF > 5% are represented with MAF in percentages and bolded.

### CYP2C19 and CYP2D6 Functional Variant Genotyping

Functional *CYP2C19* and *CYP2D6* genetic variants include: (1) variants or Null alleles that encode nonfunctional proteins; (2) variants that cause a decrease in enzyme capacity or transcription levels compared to normal alleles, but not complete lack of enzyme; and (3) variants that result in an increase in enzyme capacity or transcription levels (12). Moreover, the *CYP2D6* gene belongs to one of the most complicated and polymorphic loci in the whole of the human genome (23). Deletion of the entire gene (CYP2D6*5) and duplication/multiplication of the gene (e.g., *CYP2D6Wt*x2/N) occur frequently worldwide (12). For all genetic variants, a distinction between common (MAF > 1%) and rare (MAF < 1%) variants can be made. The frequencies of common *CYP2C19* and *CYP2D6* functional variant alleles worldwide, as previously described by (12), are listed in **Table 1**.

Currently, *CYP2C19*, *CYP2D6* and other CYP genotyping assays mostly cover only common variants, which is understandable from an economic point of view; however, there is an abundance of rare *CYP2C19* and *CYP2D6* variant alleles (24) (https://www.pharmvar.org/gene/CYP2D6). Thus, a substantial fraction of genetically caused variation in drug metabolism cannot be resolved unless extensive sequencing efforts are carried out in a psychiatric setting. The examination of big datasets from, for example, the EXAC consortium, reveals that among all mutations seen based on whole exome sequencing of > 67,000 individuals, rare mutations account for over 90% of the number of unique SNVs and 50% of the SNVs identified are seen in only one individual (24). The total contribution of rare SNVs to interindividual variation in drug response is difficult to estimate but one quarter of the total variation appears to be a realistic figure (25). This means that in clinical practice, a substantial amount of the true functional genetic variation of drug metabolizing enzymes is not detected by using the commercially available genotyping tools. The importance of rare, often *de novo*, variants contribution for explaining interindividual variation in drug pharmacokinetics is also supported by results from twin studies where the differences in pharmacokinetics of e.g. torsemide and metoprolol were found to be much less between monozygotic as compared to dizygotic twins (26); only 30%–40% of the inheritable part of this variation was attributable to genetic variants known at that time. For more than half of the genes encoding drug metabolizing enzymes (other than *CYP2C19* and *CYP2D6*) and drug transporters, rare variants account for the entire genetic variability (24). Therefore, the only robust manner to determine the true genetic variation of patients in a comprehensive manner, would appear to be the use of sequencing techniques, which is not realistic in most of clinical facilities. However, it is likely that a transition to more use of these will appear in the coming years and that this will yield more optimal tools to assist treatment personalization in psychiatry.

**Figure 1:**
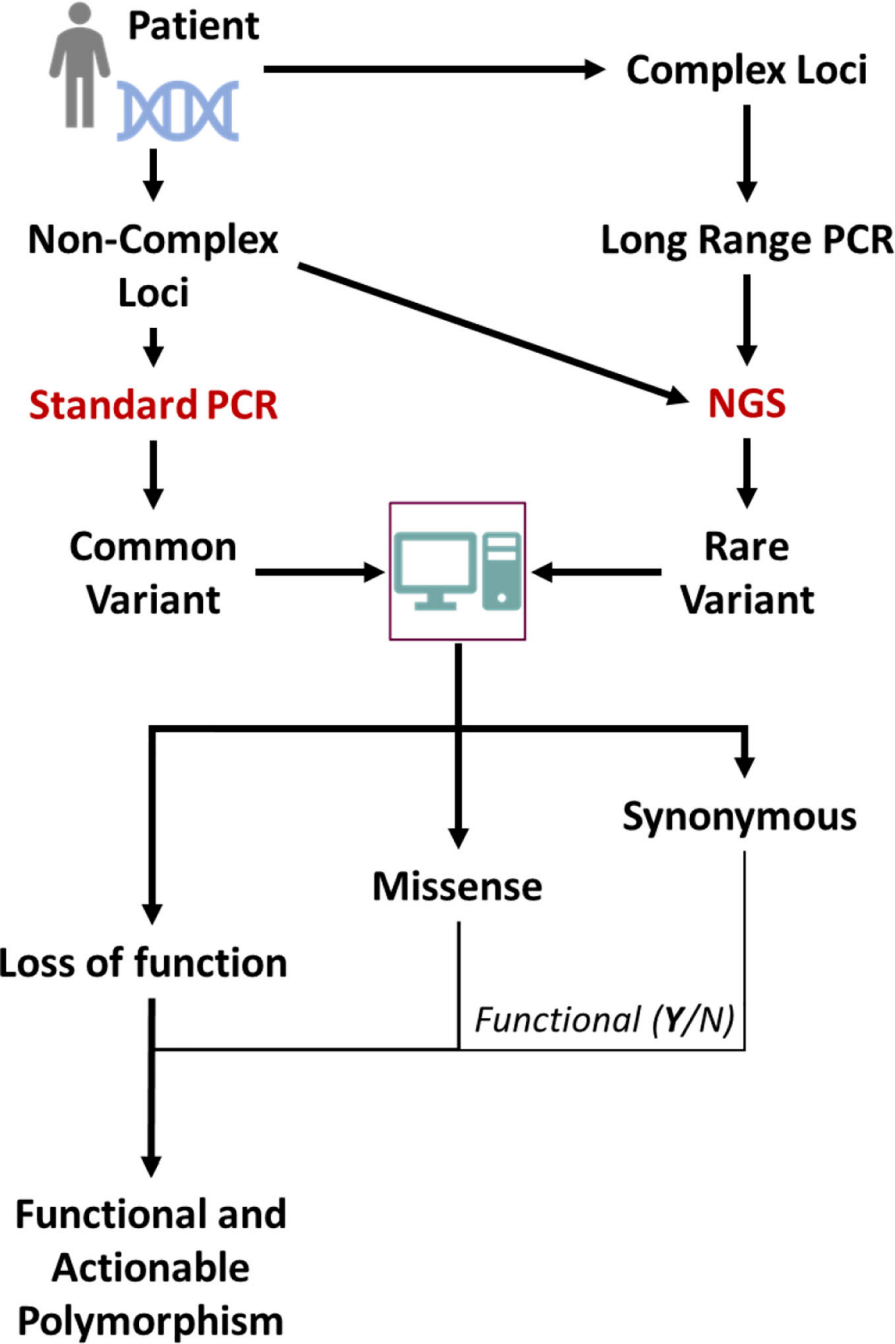
Scheme for NGS-based sequencing for determination of global genomic variations of importance for preemptive pharmacogenetics advice. Common variants might also be directly detected by using standard genotyping techniques and conventional sequencing. The gene of interest might have its origin in a complex locus like the *CYP2D* locus and for proper sequencing e.g. long range PCR methods are necessary. From the (NGS, common known mutations will be evident but also rare mutations. These can be classified by subjecting the sequences to specific algorithms that can classify the mutations according to expected functional impact.

Sequencing-based analyses present various challenges. Firstly, many pharmacogenes represent complex loci, including several genes and/or pseudogenes with very similar sequences. Conventional NGS analyses with short overlapping reads are unable to determine the genotype in such cases, as sequence similarities with neighboring genes/pseudogenes renders alignment of short sequences to the gene(s) in question very difficult or impossible. Long read sequencing or synthetic long read methods have to be employed for such loci (27). Secondly, NGS based sequencing is not able to make a firm assignment of the functionality of mutations identified, with respect to loss of function mutations and true synonymous mutations. However, relatively accurate algorithms to predict the functionality of mutations have been developed and trained on DME genes (28); the analysis workflow using such sequencing-based techniques is depicted in **Figure 1**. In the future, the application of such a broad personalized approach for analyses of genetic variation could make pharmacogenomic predictions much more attainable for individual patients.

### Phenotypic Classification Based on *CYP2C19* and *CYP2D6* Genotype

The cumulative allelic frequency of all *CYP2C19* and *CYP2D6* alleles that influence enzymatic capacity varies by ethnicity, but it is always substantial (higher than 50%); therefore, the total number of affected patients is considerable. Traditional classification usually divides patients into four groups: (**1**) poor metabolizers (PMs) are homozygous carriers of two loss-of-function alleles and their specific enzymatic capacity is completely abolished; (**2**) intermediate metabolizers (IMs) carry genotypes that are causing substantially reduced, but not absent, enzyme capacity; (**3**) normal metabolizers NMs homozygously carry normal (*Wt*) alleles and are associated with normal enzymatic capacity. NMs may also carry other genotypes as long as the enzymatic capacity is not significantly different compared with *Wt/Wt* carriers; (**4**) ultrarapid metabolizers (UM) carry genotypes linked to significantly increased enzymatic capacity compared with *Wt/Wt* carriers.

While this classification is informative and consistent with recent data, it is over-simplified, as certain genotypes that are classified within one category may carry different enzymatic capacities and the functional categorization of some specific genotypes is unclear (**Table 1**). A potential solution is the establishment of activity scores that may be used to translate highly complex *CYP2D6* or *CYP2C19* diplotype data into one value, which can be used as the estimate for phenotypic alteration (29). However, the precision of such an activity score index would require very reliable estimates of how much each variant allele affects enzymatic capacity and the activity score may also be substrate dependent. Consequently, such a classification is very simple for *Null* alleles since they completely abolish enzymatic capacity (0% activity score compared with Wt allele), but quite complicated for the other functional alleles. According to our data from 2,087 CYP2C19 genotyped patients treated with escitalopram, the CYP2C19*17 allele increases the enzymatic capacity of CYP2C19 by only approximately 20% compared with *CYP2C19Wt* (15). Similarly, in our data from 1,003 *CYP2D6* genotyped patients treated with venlafaxine, the CYP2D6*41 allele reduces the enzymatic capacity of CYP2D6 by approximately 85% compared to *CYP2D6Wt*, while CYP2D6*9 and CYP2D6*10 alleles reduce CYP2D6 enzymatic capacity by approximately 70% compared to *CYP2D6Wt* (22). **Table 2** illustrates the metabolizer categorization and activity scores based on *CYP2C19* and *CYP2D6* genotypes; however, although these estimates are quite reasonable, the activity scores may also be drug- and ethnicity-dependent and more *in vivo* studies are needed to improve the trustworthiness of the activity scores.

While associations between functional diplotype category and expected enzymatic capacity are informative, the clinically relevant information is how *CYP2C19* and *CYP2D6* genotypes translate into phenotypes such as drug concentration, and whether genotypes are associated with differential treatment outcomes. It is expected that the occurrence of *CYP2C19* and *CYP2D6* variant alleles will affect the exposure of drugs metabolized by CYP2C19 and CYP2D6, and conversely, that drugs which are specifically metabolized by CYP2C19 or CYP2D6 will be mostly affected in terms of relevant clinical phenotypes. However, specific associations and the magnitudes of such effects have to be established and quantified accordingly.

Escitalopram and sertraline are examples of drugs that are metabolized predominantly by CYP2C19 and as expected, a profound increase in escitalopram and sertraline exposure is observed in CYP2C19 PMs (15, 30). Escitalopram treated PMs are more prone to side effects and treatment dropout (15, 31); however, they also respond better to escitalopram if treatment is tolerated (31, 32). The increase in enzymatic capacity caused by the CYP2C19*17 variant seems to also affect escitalopram treatment outcome, likely by reducing the exposure (15, 33). Patients carrying CYP2C19*1/*17 and CYP2C19*17/*17 genotype had a 50% increase in treatment failure rate compared with NMs (15). Moreover, replicated findings that CYP2C19 UMs treated with escitalopram exhibit increased suicidal ideation (34, 35) indicates that distinguishing between CYP2C19 NMs and UMs is clinically relevant for the escitalopram treatment.

Risperidone is an example of a drug that is metabolized predominantly by CYP2D6, and in a study of 1,288 patients, approximately 1.4- and 1.6-fold risperidone exposure increase was observed in IMs and PMs, respectively (14). Since the therapeutic window for risperidone is quite narrow, this exposure increase is likely the reason behind the higher incidence of risperidone-associated adverse drug reactions (36) and treatment failure (14) observed among PMs compared with NMs. Furthermore, an increased treatment failure rate is also observed in Ums (14), indicating that they might be exposed to insufficient drug levels. Many other antipsychotics, such as aripiprazole and haloperidol, are metabolized by CYP2D6. Although aripiprazole metabolism is not as CYP2D6 selective as that of risperidone, CYP2D6 genotype clearly affects drug exposure for aripiprazole: a 1.6-fold increased drug exposure is observed in CYP2D6 PMs in a study of 1,334 patients (14). Since aripiprazole has a very long half-life (almost a week), the net clinical effect is significant; hence, halving the aripiprazole dose is recommended on the FDA drug label for known CYP2D6 PMs (FDA Aripiprazole).

### Translation of CYP2C19 and CYP2D6 Phenotypes Into Clinical Recommendations

It is not realistic to expect that clinicians are able to follow scientific literature on a regular basis in addition to their clinical work, and it is therefore essential to differentiate robust scientific evidence from the findings that need further validation and to translate the former into informative treatment guidelines. There is a research gap in knowledge regarding the cost-effectiveness of preemptive genotyping in psychiatry; however, it has previously been suggested that pharmacogenetic testing in psychiatry might be cost-effective if the tests became cheaper. Importantly, most of the models did not consider indirect costs, which are known to be high for psychiatric patients (37–39). In the EU, the direct and indirect costs of mental health disorders have been estimated at €798 billion and are expected to double by 2030 (40). The cost of genetic testing is dropping each year, rendering its implementation in daily psychiatric care more and more possible.

In addition, utilization of the outputs from pharmacogenetic testing can be very challenging in the complex real-life situations (41). Patients often suffer from multiple disorders and are therefore taking combinations of medications, which can influence the metabolism of the CNS active drugs. This phenomenon causes the conversion of genetic EMs into phenotypic UMs, IMs, or PMs (phenoconversion) owing to the effects of concomitant medications on enzyme inhibition and induction and this can modify clinical response to drugs. This has mostly not been taken into account in clinical studies and there is a therefore a real risk that such studies may have missed clinically relevant phenomena.

Although the scientific evidence for the above mentioned *CYP2C19* and *CYP2D6* genes is strong enough to recommend clinical application, larger international and nonindustry-funded implementation studies demonstrating feasibility and cost-effectiveness in real-world settings are lacking. Real-world settings include people that use more than one drug, which is something that the current guidelines do not yet cover. Possible obstacles in terms of feasibility include the availability of an efficient system to generate, deliver and implement genotyping in the clinical prescription of psychiatric medication. At this time, despite gene-dosing advice for many antidepressants (**Tables 3** and **4**) and antipsychotics (**Table 5**) these are all prescribed to patients with almost no preemptive genotyping or genetic analyses during treatment, not even when side effects occur or when the pharmacotherapy is inefficacious.

**Table 3:** Dosing advice of antidepressants based on CYP2C19 and CYP2D6 phenotype according to DPWG and/orCPIC; see (77).

SSRI/SNRI	Poor metabolizer	CYP2C19			CYP2D6			
		Intermediate metabolizer	Normal metabolizer	Ultrarapid metabolizer	Poor metabolizer	Intermediate metabolizer	Normal metabolizer	Ultrarapid metabolizer
Citalopram	50% of starting dose or alternative	Recommended starting dose	Recommended starting dose	Alternative drug	Recommended starting dose	Recommended starting dose	Recommended starting dose	Recommended starting dose
Escitalopram	50% of starting dose or alternative	Recommended starting dose	Recommended starting dose	Alternative drug	Recommended starting dose	Recommended starting dose	Recommended starting dose	Recommended starting dose
Fluvoxamine	Recommended starting dose	Recommended starting dose	Recommended starting dose	Recommended starting dose	25%–50% or alternative	Recommended starting dose	Recommended starting dose	
Mirtazapine	Recommended starting dose	Recommended starting dose	Recommended starting dose	Recommended starting dose				
Paroxetine	Recommended starting dose	Recommended starting dose	Recommended starting dose	Recommended starting dose	Alternative or 50%	Recommneded starting dose	Recommended starting dose	Recommended starting dose
Sertraline	50% of starting dose	Recommended starting dose	Recommended starting dose	Recommneded starting dose				
Venlafaxine					Alternative drug or adjust	Alternative drug or adjust	Recommended starting dose	Likely higher dose e.g. 150% or alternative drug

**Table 4:** Tricyclic antidepressants dosing advice based on CYP2C19 and CYP2D6 phenotype according to DPWG and/or CPIC; see (78).

**TCAs**	CYP2C19				CYP2D6				
	Poor metabolizer	Intermediate metabolizer	Normal metabolizer	Ultrarapid metabolizer	Poor metabolizer	Intermediate metabolizer	Normal metabolizer	Ultrarapid metabolizer	
Amitriptyline	Nortriptyline/ desimpramine or 50% dose reduction	Recommended starting dose	Recommended starting dose	Nortriptyline/ desimpramine or 50% dose increase	No TCA or 50%	Reduce dose by 25%	Recommended starting dose	No TCA or higher dose	
Clomipramine	Nortriptyline/ desimpramine or 50%	Recommended starting dose	Recommended starting dose	Nortriptyline/ desimpramine or 50% dose increase	No TCA or 50% dose reduction	Reduce dose by 25%	Recommended starting dose	No TCA or higher dose	
Doxepine	Nortriptyline/ desimpramine or 50%	Recommended starting dose	Recommended starting dose	Nortriptyline/ desimpramine or 50%	Reduce dose by 60%	Reduce dose by 20%	Recommended starting dose	No TCA or 100% higher dose	
Imipramine	Nortriptyline/ desimpramine or 50%	Recommended starting dose	Recommended starting dose	Nortriptyline/ desimpramine or 50%	No TCA or 70% dose reduction	No TCA or 30% dose reduction	Recommended starting dose	No TCA or 70% dose increase	
Nortriptyline		Recommended starting dose	Recommended starting dose	100%	No TCA or 60% dose reduction	No TCA or 40% dose reduction	Recommended starting dose	No TCA or 60% dose increase	

**Table 5:** Antipsychotic dosing advice based on CYP2D6 phenotype according to DPWG (https://www.pharmgkb.org/gene/PA128/guidelineAnnotation/PA166104988) according to the recent large-scale study results (14).

	CYP2D6			
	Poor metabolizer	Intermediate metabolizer	Normal metabolizer	Ultrarapid metabolizer
Aripiprazole	Reduce maximum dose by 33% (to 20 mg/day)	Reduce maximum dose by 33% (to 20 mg/day)	Recommended starting dose	Be alert for subtherapeutic drug levels (TDM) OR Select alternative drug
Risperidone	Reduce maximum dose by 33% (to 4 mg/day)	Reduce maximum dose by 33% (to 4 mg/day)	Recommended starting dose	Be alert for subtherapeutic drug levels (TDM) OR Select alternative drug
Haloperidol	Reduce the dose by 50% OR select alternative drug	Be alert to ADRs OR select alternative drug	Recommended starting dose	Be alert for subtherapeutic drug levels (TDM) OR Select alternative drug
Zuclopenthixol	Reduce the dose by 50% OR select alternative drug	Reduce the dose by 25% OR select alternative drug	Recommended starting dose	Be alert for subtherapeutic drug levels (TDM) OR Select alternative drug

### Pharmacodynamic Pharmacogenetics

Research oriented towards pharmacodynamic-related biomarkers has to date mainly focused on functional variants of genes encoding drug targets for currently available antipsychotics and antidepressants such as SERT (*SLC6A4*), NET (*SLC6A2*), *DRD2*, *HTR1A*, and *HTR2A*. In addition, genes coding pivotal proteins involved in neuroprotection, stress-response and immune system activation such as BDNF, FKPB5, CHRBP, HLA-A, and HLA-B have been considered. However, despite initial enthusiasm, most of these polymorphisms have not yet been validated for clinical utility.

One example is a polymorphism in the promoter region of the serotonin transporter (*5-HTTLPR*), which is one of the most studied genetic polymorphisms for response to antidepressants. The deletion of a 44-bp long region gives rise to a short (S)/long (L) variant in the promoter region, the short having been associated with lower levels of SERT expression (42). The long (L) variant was initially associated with better response to SSRIs (43). After 28 studies were conducted to either validate or dismiss this finding, a recent systematic review was performed and according to the meta-analysis therein, this variant did not conclusively affect SSRI treatment outcome (44). Although it may nonetheless be possible that it is relevant together with other data(45), at the present date, it is unlikely that *5-HTTLPR* genotyping, which is present in more than one third of currently available commercially assays (13) can be utilized as an effective clinical predictor for drug response. Similarly, rs7997072 (*HTR2A*) (46), rs5569 (SLC6A2) (47), and rs1360780 (FKBP5) (48) were suggested to be predictors of response to SSRIs, SNRIs, and all antidepressants admixed respectively with reasonable confidence levels and effect sizes. However, these findings were not replicated in a precise manner by the GENDEP study (49). For example, for the *HTR2A*, different markers (rs2224721 and rs9316233) in the same region of the gene (intron 2) as rs7997072 were nominally significantly associated with response to escitalopram in GENDEP study (49). It is likely that the effect of *HTR2A* polymorphism involves complex polygenic gene-environment interactions; however, these interactions are yet to be elucidated. A further example is the -141C Ins/Del polymorphism in the *DRD2* gene, which in a GWAS was associated with risk of schizophrenia and was suggested to predict response to antipsychotic therapy (50); in addition, a recent report linked the minor allele with less favorable response to clozapine (51). However, the level of significance was only marginal (*p* value between 0.05 and 0.01), suggesting that the clinical relevance of this SNP, when considered alone, is limited. Of note, CYP2C19 and CYP2D6 are both expressed in the human brain (52, 53), where they can metabolize trace amines to monoamines and affect psychiatric-relevant phenotypes and pharmacological treatment outcomes (54, 55). Substantial effort has been made to elucidate the endogenous role of these two enzymes in the brain and the impact of variant *CYP2C19* and *CYP2D6* alleles. However, it is not yet clear whether their endogenous role and local drug metabolism can affect treatment with psychiatric drugs. This topic however, is beyond the scope of this review; for further reading see (56) and. P-glycoprotein (P-gp) is a plasma membrane efflux pump encoded by the *ABCB1* gene which acts as a drug transport mechanism, actively exporting drugs from cerebrospinal fluid to blood. Many psychiatric drugs are substrates for P-gp and functional polymorphism in *ABCB1* might hypothetically change the levels of such drugs at the site of action; however, although certain variants of *ABCB1* have been associated with alterations in drug disposition and response, the results have been highly conflicting, with questionable clinical relevance (57).

Other types of polymorphic loci, such as the HLA locus, can be used for prediction of drug response and adverse drug reactions. For example, the HLA-B*1502 allele is connected with an increased risk of developing Stevens-Johnson syndrome (SJS) and its related disease, toxic epidermal necrolysis (TEN) during carbamazepine or oxcarbazepine treatment (58). HLA-B*1502 is common among East Asians (6.9%, apart from Japanese at <1% and Korean at <2.5%); Oceanic (5.4%); and South/Central Asian (4.6%) ethnic groups have relatively high frequencies of HLA-B*1502 (58). Among carbamazepine treated patients, HLA-B*1502 increases the risk of SJS/TEN hundredfold (OR: 113.4; 95% CI: 51.2-251.0) (59). While the positive predictive value of HLA-B*1502 genotype is much less for oxcarbazepine then for carbamazepine, the negative predictive value for both is nearly 100% in South East Asian populations (60). In a trial of 4,877 patients in Taiwan, HLA-B*1502 genotyping completely prevented SJS/TEN from occurring, while the estimated historical incidence of carbamazepine-induced SJS-TEN (0.23%) would translate into approximately 10 cases among study subjects (61). As a result of this evidence, the FDA introduced a black-box warning on the carbamazepine drug label and proscribed mandatory genotyping for the HLA-B*1502 in patients with documented Asian origin (https://www.accessdata.fda.gov/drugsatfda_docs/label/2009/016608s101,018281s048lbl.pdf). CPIC provides guidelines for HLA-B*1502 and HLA-A*31:01 variants for all carbamazepine and oxcarbazepine-treated patients, irrespective of region of origin or ethnic group (58). This example illustrates how drug labelling and clinical guidelines follow, once the clinical studies are able to provide firm and robust evidence about the usefulness of preemptive genotyping.

In the present situation, where commercial companies specialized in the sale of genetic test results for profit have proliferated, there is an increasing risk of advertisements relatied to offering nonvalidated pharmacodynamic- and pharmacokinetic-based genotypes directly to consumers (patients). In fact, for inadequately informed clinicians, such genetic testing may actually cause more harm than benefit. One of the examples that illustrates this possibility is a recently published case report describing a patient whose family and outpatient care provider received the information that he was a -141Cdel carrier. Since the genetic test results implied that the patient was expected to poorly respond to clozapine, objection was raised when switching the antipsychotic to clozapine was suggested to the patient. However, since the symptom level was severe, after consulting with the hospital medical director, clozapine was nonetheless administered to the patient and this had a remarkably good and rapid effect (62). As previously mentioned, the *DRD2* -141C ins/del is very far from a validated biomarker for clozapine response and is seldom present on any of the commercially available assays (13). This case therefore illustrates the danger of testing using insufficiently validated genetic markers and provides an example of how such testing can cause a setback in the clinician-patient relationship. We therefore conclude that healthcare providers who order and deal with pharmacogenetic testing should have a moral and legal responsibility to educate themselves and their patients about pharmacogenetic testing and its limitations. Furthermore, regulatory and legislative frameworks should be developed and implemented to guide this process.

## Discussion

The number of drugs carrying pharmacogenomic labels continues to increase (6, 63), while in contrast, the chance of discovering more blockbuster drugs is decreasing. Therefore the advancement of pharmacotherapy in psychiatry and in general in the forseeable future will likely be dependent on adequate drug treatment personalization (64). One example is the treatment of cystic fibrosis, where genotyping of the *CFTR* gene directly impacts the likelihood for successful treatment using *CFTR* modulators (65). Guidelines for pharmacogenomic work during drug development have been developed and are instrumental tools for the pharmaceutical industry (https://www.ema.europa.eu/en/documents/scientific-guideline/guideline-good-pharmacogenomic-practice-first-version_en.pdf), particuarly in oncology.

With respect to psychiatry, the HLA markers described above are relevant for mood stabilizers carbamazepine and oxcarbamazepine. While genotyping to avoid life-threatening adverse drug reactions is mandatory for the HLA markers, pharmacoeconomic analyses provide support for the cost-effectiveness of other pharmacogenetic-based treatment decisions, especially in the case of *CYP2D6* (66, 67). Currently, it seems evident that, out of many proposed markers, only genetic variants located in the *CYP2C19* and *CYP2D6* genes are of value for optimizing antipsychotic and antidepressant drug treatment. These genes were consistently found to influence pharmacotherapy response in seven randomized controlled trials when genotyping was performed prospectively (68–74). While genotyping pharmacodynamic genes such as *DRD2* is not at this time sufficiently supported by research results, it is possible that machine learning of multivariate data including pharmacokinetic and pharmacodynamic genes may produce associations of interest.

Variants in pharmacogenes often have low allele frequencies and thus for good power, thousands of patients, or meta-analyses in which variants are grouped together into functional categories, are required for robust results. While neuropsychopharmacogenomic clinical trials including a health economic component could strengthen the evidence for cost savings, in general there is a need for a larger, nonindustry sponsored prospective trials. For some gene-drug pairs, some might argue that the strength of the evidence is sufficient and that such a clinical trial might be unnecessary and unethical. For most of the drugs however, the situation is not quite clear and further large analyses are indicated; moreover, even for the validated drug-gene pairs, some would argue that clinical evidence are still incomplete (16). It is also possible that further retrospective analyses of relevant clinical trials already conducted, especially where cost-related data are available, could generate informative health economic data. Certainly, pharmacogenomic analyses should incorporate comprehensive genomic analysis (sequencing) and also carefully consider factors that may influence the findings, such as comedications and pathophysiolgical factors.

Therapeutic drug monitoring (TDM) of drug plasma levels has been used in psychiatry for a long time, in particular for tricyclic antidepressants, lithium, and clozapine. TDM captures all factors of interindividual variability in drug metabolism, while genotyping can offer only a solid prediction of patient metabolic capacity (19); however, genotyping is possible before drug treatment is initiated, whereas TDM can only be performed when the drug levels reach steady-state and when the patient is possibly already exposed to adverse drug reactions. Therefore, in an ideal constellation, a psychiatrist would take into consideration all available genetic, somatic, dietary and environmental parameters to make the best possible drug and dose selection at initiation of therapy for each individual patient, and once the drug levels reach steady-state, TDM would be utilized to ascertain whether such an educated decision was indeed optimal. Since the efficacy rate is only 30%–40% for antidepressants and antipsychotics (75, 76) despite TDM, there is clearly room for an improvement in approach.

## Conclusion

To conclude, there are good pharmacogenetic clinical recommendations for a wide selection of psychopharmacological agents based on *CYP2C19* and *CYP2D6* functional diplotypes. In addition, recent adequately-powered studies (n > 1,000) have yielded more clinically useful information and support the clinical utility of preemptive pharmacogenomics for specific gene-drug pairs. To extend the knowledge beyond these gene-drug pairs, further large-scale prospective randomized trials having a sequencing-based genetic approach and/or reanalyses of studies with economic data are needed. At this point, there would appear to be sufficient data to support implementation of pharmacogenomics in to daily psychiatric practice in patients experiencing side effects and/or inefficacy (**Tables 1**–**3**). Finally, it is crucial that clinicians are adequately educated in the field of pharmacogenetics and equipped with adequate guidelines and decision support tools to interpret genotyping results and translate them into the clinical setting.

## Author Contributions

RW drafted pharmacokinetics—clinical part. KA drafted the discussion KA and MJ drafted pharmacodynamics part. MI-S drafted the introduction and conclusion. MI-S and MJ drafted pharmacogenetics—genetics part. MJ drafted pharmacokinetics introduction part and pharmacokinetics—phenotypes part. All authors edited and approved all parts.

## Funding

2017 Koers 2017/2018 Internal funding Erasmus MC to start Outpatient clinic Pharmacogenetics in Psychiatry, Instigator and PI to RW 2017 NL63514.019.17 Pharmacogenetics to improve personalized antidepressant dosing in patients with severe depression: a randomized controlled trial using tricyclic antidepressants. Co-applicant (PI ErasmusMC) to RvW 2016 Project 42653483 for a Dutch Guideline in Implementation of pharmacogenetics in psychiatry SKMS (Stichting Kwaliteitsgelden Medisch Specialisten), Instigator, and Chairperson to RW 2019 Phillip Morris grant - Utility of CYP2C19 genotyping and therapeutic drug monitoring in the personalization of escitalopram therapy to MJ.

## Conflict of Interest

KA has received the AmpliChip CYP450 Test and associated research support from Roche Molecular Systems, and has acted in a consulting capacity for companies including Bristol-Myers Squibb and Otsuka Pharmaceuticals Ltd, Otsuka Canada Pharmaceuticals Inc., Lundbeck, and HLS Therapeutics. She has also received research grants from companies including Bristol-Myers Squibb and Otsuka Pharmaceuticals, Johnson and Johnson Research and Development, and Jannsen Inc. Canada. RW is a medical consultant at Chipsoft (Electronic patient files, Netherlands) and a founder of Outpatient Clinic Pharmacogenetics Parnassia Psychiatric Institute.

The remaining authors declare that the research was conducted in the absence of any commercial or financial relationships that could be construed as a potential conflict of interest.

## References

[1] American_Psychiatric_Association Diagnostic and statistical manual of mental disorders. 5th ed. Arlington, VA: American Psychiatric Publishing (2013).

[2] Cipriani A Furukawa TA Salanti G Chaimani A Atkinson LZ Ogawa Y Comparative efficacy and acceptability of 21 antidepressant drugs for the acute treatment of adults with major depressive disorder: a systematic review and network meta-analysis. Lancet (2018) 391(10128):1357–66. 10.1016/S0140-6736(17)32802-7 PMC5889788 29477251

[3] Leucht S Cipriani A Spineli L Mavridis D Orey D Richter F Comparative efficacy and tolerability of 15 antipsychotic drugs in schizophrenia: amultiple-treatments meta-analysis. Lancet 2013; 382 (9896) 382:951–62. 10.1016/S0140-6736(13)60733-3 23810019

[4] Kirsch I Deacon BJ Huedo-Medina TB Scoboria A Moore TJ Johnson BT Initial severity and antidepressant benefits: a meta-analysis of data submitted to the food and drug administration. PloS Med (2008) 5(2):e45. 10.1371/journal.pmed.0050045 18303940 PMC2253608

[5] Lauschke VM Ingelman-Sundberg M Prediction of drug response and adverse drug reactions: from twin studies to nextgeneration sequencing. Eur J Pharm Sci (2019) 130:65–77. 10.1016/j.ejps.2019.01.024 30684656

[6] Lauschke VM Zhou Y Ingelman-Sundberg M Novel genetic and epigenetic factors of importance for inter-individual differences in drug disposition, response and toxicity. Pharmacol Ther (2019) 197:122–52. 10.1016/j.pharmthera.2019.01.002 PMC6527860 30677473

[7] Eichelbaum M Ingelman-Sundberg M Evans WE Pharmacogenomics and individualized drug therapy. Annu Rev Med (2006) 57:119–37. 10.1146/annurev.med.56.082103.104724 16409140

[8] Shekhani R Steinacher L Swen JJ Ingelman-Sundberg M Evaluation of current regulation and guidelines of pharmacogenomic drug labels: opportunities for improvements. Clin Pharmacol Ther (2019) in press. 10.1002/cpt.1720 PMC7232863 31715018

[9] Koutsilieri S Tzioufa F Sismanoglou DC Patrinos GP Unveiling the guidance heterogeneity for genome-informed drug treatment interventions among regulatory bodies and research consortia. Pharmacol Res (2019) in press. 10.1016/j.phrs.2019.104590 31830522

[10] Kashoki M Hanaizi Z Yordanova S Vesely R Bouygues C Llinares J A Comparison of EMA and FDA decisions for new drug marketing applications 2014-2016: concordance, discordance, and why. Clin Pharmacol Ther (2020) 107(1):195–202. 10.1002/cpt.1565 31306483 PMC6977394

[11] Ingelman-Sundberg M Translation of pharmacogenomic drug labels into the clinic. current problems. Pharmacol Res (2019) in press. 10.1016/j.phrs.2019.104620 31899313

[12] Zhou Y Ingelman-Sundberg M Lauschke VM Worldwide distribution of cytochrome P450 alleles: a meta-analysis of population-scale sequencing projects. Clin Pharmacol Ther (2017) 102(4):688–700. 10.1002/cpt.690 28378927 PMC5600063

[13] Bousman CA Dunlop BW Genotype, phenotype, and medication recommendation agreement among commercial pharmacogenetic-based decision support tools. Pharmacogenomics J (2018) 18(5):613–22. 10.1038/s41397-018-0027-3 29795409

[14] Jukic MM Smith RL Haslemo T Molden E Ingelman-Sundberg M Effect of CYP2D6 genotype on exposure and efficacy of risperidone and aripiprazole: a retrospective, cohort study. Lancet Psychiatry (2019) 6(5):418–26. 10.1016/S2215-0366(19)30088-4 31000417

[15] Jukic MM Haslemo T Molden E Ingelman-Sundberg M Impact of CYP2C19 genotype on escitalopram exposure and therapeutic failure: a retrospective study based on 2,087 patients. Am J Psychiatry (2018) 175(5):463–70. 10.1176/appi.ajp.2017.17050550 29325448

[16] Zeier Z Carpenter LL Kalin NH Rodriguez CI McDonald WM Widge AS Clinical implementation of pharmacogenetic decision support tools for antidepressant drug prescribing. Am J Psychiatry (2018) 175(9):873–86. 10.1176/appi.ajp.2018.17111282 PMC6774046 29690793

[17] Hauser AS Chavali S Masuho I Jahn LJ Martemyanov KA Gloriam DE Pharmacogenomics of GPCR drug targets. Cell (2018) 172(1-2):41–54 e19. 10.1016/j.cell.2017.11.033 29249361 PMC5766829

[18] Shimada T Yamazaki H Mimura M Inui Y Guengerich FP Interindividual variations in human liver cytochrome P-450 enzymes involved in the oxidation of drugs, carcinogens and toxic chemicals: studies with liver microsomes of 30 Japanese and 30 Caucasians. J Pharmacol Exp Ther (1994) 270(1):414–23. 8035341

[19] Hiemke C Bergemann N Clement HW Conca A Deckert J Domschke K consensus guidelines for therapeutic drug monitoring in neuropsychopharmacology: update 2017. Pharmacopsychiatry (2018) 51(1-02):9–62. 10.1055/s-0043-116492 28910830

[20] Pardinas AF Nalmpanti M Pocklington AJ Legge SE Medway C King A Pharmacogenomic variants and drug interactions identified through the genetic analysis of clozapine metabolism. Am J Psychiatry (2019) 176(6):477–86. 10.1176/appi.ajp.2019.18050589 30922102

[21] Lamba J Lamba V Schuetz E Genetic variants of PXR (NR1I2) and CAR (NR1I3) and their implications in drug metabolism and pharmacogenetics. Curr Drug Metab (2005) 6(4):369–83. 10.2174/1389200054633880 16101575

[22] Haslemo T Eliasson E Jukic MM Ingelman-Sundberg M Molden E Significantly lower CYP2D6 metabolism measured as the O/N-desmethylvenlafaxine metabolic ratio in carriers of CYP2D6*41 versus CYP2D6*9 or CYP2D6*10: a study on therapeutic drug monitoring data from 1003 genotyped Scandinavian patients. Br J Clin Pharmacol (2019) 85(1):194–201. 10.1111/bcp.13788 30312494 PMC6303206

[23] Gaedigk A Complexities of CYP2D6 gene analysis and interpretation. Int Rev Psychiatry (2013) 25(5):534–53. 10.3109/09540261.2013.825581 24151800

[24] Ingelman-Sundberg M Mkrtchian S Zhou Y Lauschke VM Integrating rare genetic variants into pharmacogenetic drug response predictions. Hum Genomics (2018) 12(1):26. 10.1186/s40246-018-0157-3 29793534 PMC5968569

[25] Ingelman-Sundberg M Lauschke VM Current statistical metrics are pragmatic measures to compare the predictive quality of preclinical assays. Toxicological Sci (2018) 165(1):4–5. 10.1093/toxsci/kfy160 30063796

[26] Matthaei J Brockmoller J Tzvetkov MV Sehrt D Sachse-Seeboth C Hjelmborg JB Heritability of metoprolol and torsemide pharmacokinetics. Clin Pharmacol Ther (2015) 98(6):611–21. 10.1002/cpt.258 26344676

[27] Lauschke VM Milani L Ingelman-Sundberg M Pharmacogenomic biomarkers for improved drug therapy-recent progress and future developments. AAPS J (2017) 20(1):4. 10.1208/s12248-017-0161-x 29181807

[28] Zhou Y Mkrtchian S Kumondai M Hiratsuka M Lauschke VM An optimized prediction framework to assess the functional impact of pharmacogenetic variants. Pharmacogenomics J (2019) 19(2):115–26. 10.1038/s41397-018-0044-2 PMC6462826 30206299

[29] Gaedigk A Dinh JC Jeong H Prasad B Leeder JS Ten years' experience with the cyp2d6 activity score: a perspective on future investigations to improve clinical predictions for precision therapeutics. J Personalized Med (2018) 8(2):E15. 10.3390/jpm8020015 PMC6023391 29673183

[30] Braten LS Haslemo T Jukic MM Ingelman-Sundberg M Molden E Kringen MK Impact of CYP2C19 genotype on sertraline exposure in 1200 Scandinavian patients. Neuropsychopharmacology (2020) 45(3):570–6. 10.1038/s41386-019-0554-x PMC6969041 31649299

[31] Fabbri C Tansey KE Perlis RH Hauser J Henigsberg N Maier W Effect of cytochrome CYP2C19 metabolizing activity on antidepressant response and side effects: Meta-analysis of data from genome-wide association studies. Eur neuropsychopharmacology (2018) 28(8):945–54. 10.1016/j.euroneuro.2018.05.009 30135031

[32] Mrazek DA Biernacka JM O'Kane DJ Black JL Cunningham JM Drews MS CYP2C19 variation and citalopram response. Pharmacogenetics Genomics (2011) 21(1):1–9. 10.1097/FPC.0b013e328340bc5a 21192344 PMC3090085

[33] Huezo-Diaz P Perroud N Spencer EP Smith R Sim S Virding S CYP2C19 genotype predicts steady state escitalopram concentration in GENDEP. J Psychopharmacol (2012) 26(3):398–407. 10.1177/0269881111414451 21926427

[34] Jukic MM Opel N Strom J Carrillo-Roa T Miksys S Novalen M Elevated CYP2C19 expression is associated with depressive symptoms and hippocampal homeostasis impairment. Mol Psychiatry (2017) 22(8):1155–63. 10.1038/mp.2016.204 27895323

[35] Rahikainen AL Vauhkonen P Pett H Palo JU Haukka J Ojanpera I Completed suicides of citalopram users-the role of CYP genotypes and adverse drug interactions. Int J legal Med (2019) 133(2):353–63. 10.1007/s00414-018-1927-0 30173302

[36] de Leon J Susce MT Pan RM Fairchild M Koch WH Wedlund PJ The CYP2D6 poor metabolizer phenotype may be associated with risperidone adverse drug reactions and discontinuation. J Clin Psychiatry (2005) 66(1):15–27. 10.4088/JCP.v66n0103 15669884

[37] Aitchison KJ Kerwin RW Cost-effectiveness of clozapine. A UK clinic-based study. Br J Psychiatry (1997) 171:125–30. 10.1192/bjp.171.2.125 9337946

[38] Berm EJ Hak E Postma M Boshuisen M Breuning L Brouwers JR Effects and cost-effectiveness of pharmacogenetic screening for CYP2D6 among older adults starting therapy with nortriptyline or venlafaxine: study protocol for a pragmatic randomized controlled trial (CYSCEtrial). Trials (2015) 16:37. 10.1186/s13063-015-0561-0 25636328 PMC4328880

[39] Perlis RH Patrick A Smoller JW Wang PS When is pharmacogenetic testing for antidepressant response ready for the clinic? a cost-effectiveness analysis based on data from the STAR*D study. Neuropsychopharmacology (2009) 34(10):2227–36. 10.1038/npp.2009.50 PMC3312011 19494805

[40] Gustavsson A Svensson M Jacobi F Allgulander C Alonso J Beghi E Cost of disorders of the brain in Europe 2010. Eur neuropsychopharmacology (2011) 21(10):718–79. 10.1016/j.euroneuro.2011.08.008 21924589

[41] Shah RR Smith RL Addressing phenoconversion: the Achilles' heel of personalized medicine. Br J Clin Pharmacol (2015) 79(2):222–40. 10.1111/bcp.12441 PMC4309629 24913012

[42] Murphy DL Lesch KP Targeting the murine serotonin transporter: insights into human neurobiology. Nat Rev Neurosci (2008) 9(2):85–96. 10.1038/nrn2284 18209729

[43] Smeraldi E Zanardi R Benedetti F Di Bella D Perez J Catalano M Polymorphism within the promoter of the serotonin transporter gene and antidepressant efficacy of fluvoxamine. Mol Psychiatry (1998) 3(6):508–11. 10.1038/sj.mp.4000425 9857976

[44] Culverhouse RC Saccone NL Horton AC Ma Y Anstey KJ Banaschewski T Collaborative meta-analysis finds no evidence of a strong interaction between stress and 5-HTTLPR genotype contributing to the development of depression. Mol Psychiatry (2018) 23(1):133–42. 10.1038/mp.2017.44 PMC5628077 28373689

[45] Keers R Uher R Huezo-Diaz P Smith R Jaffee S Rietschel M Interaction between serotonin transporter gene variants and life events predicts response to antidepressants in the GENDEP project. Pharmacogenomics J (2011) 11(2):138–45. 10.1038/tpj.2010.14 20212518

[46] McMahon FJ Buervenich S Charney D Lipsky R Rush AJ Wilson AF Variation in the gene encoding the serotonin 2A receptor is associated with outcome of antidepressant treatment. Am J Hum Genet (2006) 78(5):804–14. 10.1086/503820 PMC1474035 16642436

[47] Kim H Lim SW Kim S Kim JW Chang YH Carroll BJ Monoamine transporter gene polymorphisms and antidepressant response in koreans with late-life depression. Jama (2006) 296(13):1609–18. 10.1001/jama.296.13.1609 17018806

[48] Binder EB Salyakina D Lichtner P Wochnik GM Ising M Putz B Polymorphisms in FKBP5 are associated with increased recurrence of depressive episodes and rapid response to antidepressant treatment. Nat Genet (2004) 36(12):1319–25. 10.1038/ng1479 15565110

[49] Uher R Huezo-Diaz P Perroud N Smith R Rietschel M Mors O Genetic predictors of response to antidepressants in the GENDEP project. Pharmacogenomics J (2009) 9(4):225–33. 10.1038/tpj.2009.12 19365399

[50] Zhang JP Lencz T Malhotra AK D2 receptor genetic variation and clinical response to antipsychotic drug treatment: a meta-analysis. Am J Psychiatry (2010) 167(7):763–72. 10.1176/appi.ajp.2009.09040598 PMC2896457 20194480

[51] Huang E Maciukiewicz M Zai CC Tiwari AK Li J Potkin SG Preliminary evidence for association of genome-wide significant DRD2 schizophrenia risk variant with clozapine response. Pharmacogenomics (2016) 17(2):103–9. 10.2217/pgs.15.155 26666695

[52] Miksys S Rao Y Hoffmann E Mash DC Tyndale RF Regional and cellular expression of CYP2D6 in human brain: higher levels in alcoholics. J Neurochem (2002) 82(6):1376–87. 10.1046/j.1471-4159.2002.01069.x 12354285

[53] Aitchison K Datla K Rooprai H Fernando J Dexter D Regional distribution of clomipramine and desmethylclomipramine in rat brain and peripheral organs on chronic clomipramine administration. J Psychopharmacol (2010) 24(8):1261–8. 10.1177/0269881109105789 19553387

[54] Niwa T Shizuku M Yamano K Effect of genetic polymorphism on the inhibition of dopamine formation from p-tyramine catalyzed by brain cytochrome P450 2D6. Arch Biochem Biophys (2017) 620:23–7. 10.1016/j.abb.2017.03.009 28347660

[55] Yu AM Idle JR Herraiz T Kupfer A Gonzalez FJ Screening for endogenous substrates reveals that CYP2D6 is a 5-methoxyindolethylamine O-demethylase. Pharmacogenetics (2003) 13(6):307–19. 10.1097/00008571-200306000-00002 12777961

[56] Ingelman-Sundberg M Persson A Jukic MM Polymorphic expression of CYP2C19 and CYP2D6 in the developing and adult human brain causing variability in cognition, risk for depression and suicide: the search for the endogenous substrates. Pharmacogenomics (2014) 15(15):1841–4. 10.2217/pgs.14.151 25495406

[57] Wolking S Schaeffeler E Lerche H Schwab M Nies AT Impact of genetic polymorphisms of ABCB1 (MDR1, P-Glycoprotein) on drug disposition and potential clinical implications: update of the literature. Clin Pharmacokinet (2015) 54(7):709–35. 10.1007/s40262-015-0267-1 25860377

[58] Phillips EJ Sukasem C Whirl-Carrillo M Muller DJ Dunnenberger HM Chantratita W Clinical pharmacogenetics implementation consortium guideline for HLA genotype and use of carbamazepine and oxcarbazepine: 2017 Update. Clin Pharmacol Ther (2018) 103(4):574–81. 10.1002/cpt.1004 PMC5847474 29392710

[59] Yip VL Marson AG Jorgensen AL Pirmohamed M Alfirevic A HLA genotype and carbamazepine-induced cutaneous adverse drug reactions: a systematic review. Clin Pharmacol Ther (2012) 92(6):757–65. 10.1038/clpt.2012.189 23132554

[60] Chen CB Hsiao YH Wu T Hsih MS Tassaneeyakul W Jorns TP Risk and association of HLA with oxcarbazepine-induced cutaneous adverse reactions in Asians. Neurology (2017) 88(1):78–86. 10.1212/WNL.0000000000003453 27913699

[61] Chen P Lin JJ Lu CS Ong CT Hsieh PF Yang CC Carbamazepine-induced toxic effects and HLA-B*1502 screening in Taiwan. New Engl J Med (2011) 364(12):1126–33. 10.1056/NEJMoa1009717 21428768

[62] Rahman T Ash DM Lauriello J Rawlani R Misleading guidance from pharmacogenomic testing. Am J Psychiatry (2017) 174(10):922–4. 10.1176/appi.ajp.2017.16121353 28965468

[63] Ehmann F Caneva L Prasad K Paulmichl M Maliepaard M Llerena A Pharmacogenomic information in drug labels: european medicines agency perspective. Pharmacogenomics J (2015) 15(3):201–10. 10.1038/tpj.2014.86 25707393

[64] Ingelman-Sundberg M Pharmacogenomic biomarkers for prediction of severe adverse drug reactions. New Engl J Med (2008) 358(6):637–9. 10.1056/NEJMe0708842 18256400

[65] Paranjape SM Mogayzel PJ Jr. Cystic fibrosis in the era of precision medicine. Paediatr Respir Rev (2018) 25:64–72. 10.1016/j.prrv.2017.03.001 28372929

[66] Alagoz O Durham D Kasirajan K Cost-effectiveness of one-time genetic testing to minimize lifetime adverse drug reactions. Pharmacogenomics J (2016) 16(2):129–36. 10.1038/tpj.2015.39 25987241

[67] Sluiter RL Janzing JGE van der Wilt GJ Kievit W Teichert M An economic model of the cost-utility of pre-emptive genetic testing to support pharmacotherapy in patients with major depression in primary care. Pharmacogenomics J (2019) 19(5):480–9. 10.1038/s41397-019-0070-8 30647446

[68] Hall-Flavin DK Winner JG Allen JD Jordan JJ Nesheim RS Snyder KA Using a pharmacogenomic algorithm to guide the treatment of depression. Transl Psychiatry (2012) 2:e172. 10.1038/tp.2012.99 23047243 PMC3565829

[69] Hall-Flavin DK Winner JG Allen JD Carhart JM Proctor B Snyder KA Utility of integrated pharmacogenomic testing to support the treatment of major depressive disorder in a psychiatric outpatient setting. Pharmacogenet Genomics (2013) 23(10):535–48. 10.1097/FPC.0b013e3283649b9a 24018772

[70] Winner JG Carhart JM Altar CA Allen JD Dechairo BM A prospective, randomized, double-blind study assessing the clinical impact of integrated pharmacogenomic testing for major depressive disorder. Discovery Med (2013) 16(89):219–27. 24229738

[71] Singh AB Improved antidepressant remission in major depression *via a* pharmacokinetic pathway polygene pharmacogenetic report. Clin Psychopharmacol neuroscience (2015) 13(2):150–6. 10.9758/cpn.2015.13.2.150 PMC4540033 26243841

[72] Perez V Salavert A Espadaler J Tuson M Saiz-Ruiz J Saez-Navarro C Efficacy of prospective pharmacogenetic testing in the treatment of major depressive disorder: results of a randomized, double-blind clinical trial. BMC Psychiatry (2017) 17(1):250. 10.1186/s12888-017-1412-1 28705252 PMC5513031

[73] Bradley P Shiekh M Mehra V Vrbicky K Layle S Olson MC Improved efficacy with targeted pharmacogenetic-guided treatment of patients with depression and anxiety: a randomized clinical trial demonstrating clinical utility. J Psychiatr Res (2018) 96:100–7. 10.1016/j.jpsychires.2017.09.024 28992526

[74] Greden JF Parikh SV Rothschild AJ Thase ME Dunlop BW DeBattista C Impact of pharmacogenomics on clinical outcomes in major depressive disorder in the GUIDED trial: a large, patient- and rater-blinded, randomized, controlled study. J Psychiatr Res (2019) 111:59–67. 10.1016/j.jpsychires.2019.01.003 30677646

[75] Walker E Kestler L Bollini A Hochman KM Schizophrenia: etiology and course. Annu Rev Psychol (2004) 55:401–30. 10.1146/annurev.psych.55.090902.141950 14744221

[76] Pampallona S Bollini P Tibaldi G Kupelnick B Munizza C Combined pharmacotherapy and psychological treatment for depression: a systematic review. Arch Gen Psychiatry (2004) 61(7):714–9. 10.1001/archpsyc.61.7.714 15237083

[77] Hicks JK Bishop JR Sangkuhl K Muller DJ Ji Y Leckband SG Clinical Pharmacogenetics Implementation Consortium (CPIC) Guideline for CYP2D6 and CYP2C19 genotypes and dosing of selective serotonin reuptake Inhibitors. Clin Pharmacol Ther (2015) 98(2):127–34. 10.1002/cpt.147 PMC4512908 25974703

[78] Hicks JK Sangkuhl K Swen JJ Ellingrod VL Muller DJ Shimoda K Clinical pharmacogenetics implementation consortium guideline (CPIC) for CYP2D6 and CYP2C19 genotypes and dosing of tricyclic antidepressants: 2016 update. Clin Pharmacol Ther (2017) 102(1):37–44. 10.1002/cpt.597 27997040 PMC5478479

